# Oxygen consumption rate of flatworms under the influence of wake‐ and sleep‐promoting neurotransmitters

**DOI:** 10.1002/jez.2828

**Published:** 2024-05-27

**Authors:** Shauni E. T. Omond, Robert G. Barker, Oana Sanislav, Paul R. Fisher, Sarah J. Annesley, John A. Lesku

**Affiliations:** ^1^ School of Agriculture, Biomedicine and Environment La Trobe University Melbourne Australia; ^2^ Present address: The Brigham and Women's Hospital, Harvard Medical School Boston USA

**Keywords:** circadian, dopamine, *Dugesia*, GABA, *Girardia*, planarian, platyhelminthes

## Abstract

Flatworms are among the best studied animal models for regeneration; however, they also represent an emerging opportunity to investigate other biological processes as well. For instance, flatworms are nocturnal and sleep during the day, a state that is regulated by sleep/wake history and the action of the sleep‐promoting neurotransmitter gamma‐aminobutyric acid (or GABA). Sleep is widespread across the animal kingdom, where it serves many nonexclusive functions. Notably, sleep saves energy by reducing metabolic rate and by not doing something more energetically taxing. Whether the conservation of energy is apparent in sleeping flatworms is unclear. We measured the oxygen consumption rate (OCR) of flatworms dosed with either (*1*) GABA (*n* = 29) which makes flatworms inactive or (*2*) dopamine (*n* = 20) which stimulates flatworms to move, or (*3*) day and night neurotransmitter‐free controls (*n* = 28 and 27, respectively). While OCR did not differ between the day and night, flatworms treated with GABA used less oxygen than those treated with dopamine, and less than the day‐time control. Thus, GABA affected flatworm physiology, ostensibly by enforcing energy‐conserving sleep. Evidence that dopamine increased metabolism was less strong. This work broadens our understanding of flatworm physiology and expands the phylogenetic applicability of energy conservation as a function of sleep.

## Research Highlights

While flatworms are known to sleep, it has been unclear whether restful flatworms reap the metabolic benefits of this quiescent state. Using neurotransmitters that regulate sleep and wakefulness, we show that sleep‐promoting gamma‐aminobutyric acid reduces oxygen consumption in flatworms.

## Graphical abstract

In the free‐living platyhelminth flatworm, *Girardia tigrina*, the sleep‐promoting neurotransmitter, gamma‐aminobutyric acid (or GABA), decreases oxygen consumption rate relative to the wake‐promoting neurotransmitter, dopamine, and also a neurotransmitter‐free control. This metabolic rate study builds on the behavioral and biochemical characterization of sleep in flatworms.

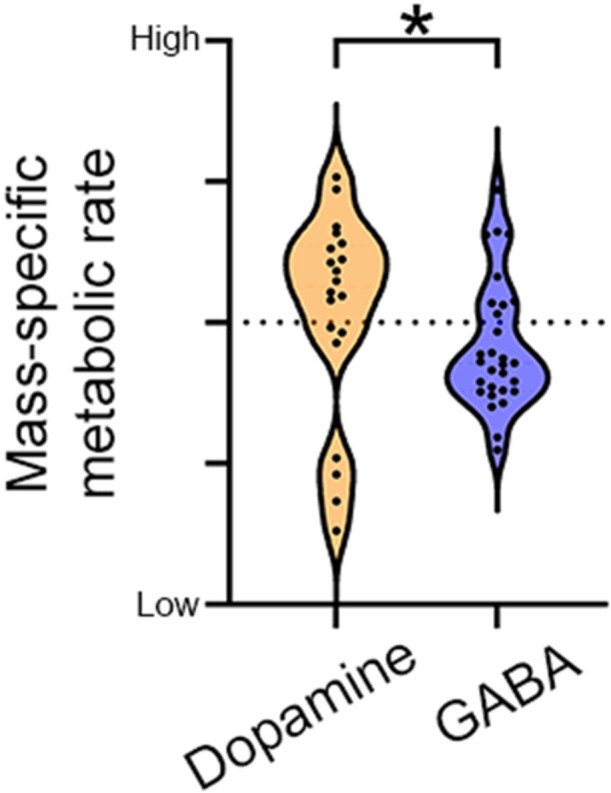

## 1 INTRODUCTION

Metabolism enables all physiological processes, in turn allowing for behavioral and ecological interactions. Yet, the amount of energy an organism obtains, assimilates, and stores is finite, such that animals are well‐motivated to seek energetic efficiencies wherever possible. One such solution is sleeping. Despite the dangers inherent in being less aware of one's surroundings (Lima et al., 2005), sleep must serve essential functions to have persisted across evolutionary time (Rattenborg & Ungurean, 2023; Zaid et al., 2024). Accordingly, one long‐standing idea for sleep function relates to energy conservation (Berger & Phillips, 1995; Siegel, 2005; Zepelin & Rechtschaffen, 1974). Sleeping saves energy by reducing metabolic rate and by enforcing inactivity (Lesku & Schmidt, 2022; Siegel, 2009). For example, lean garden warblers (*Sylvia borin*), depauperate of energy stores, lower their metabolic rate during sleep and do so with their head tucked into the feathers over their back to conserve heat; this strategy stands in contrast to one adopted by more robust warblers that maintain a higher metabolic rate and sleep with their beak exposed, facing forward (Ferretti et al., 2019). Energy savings during sleep have been reported in other animals as well, including mammals (Sharma & Kavuru, 2010), sharks (Kelly et al., 2022), and fruit flies (Brown et al., 2022; Stahl et al., 2017). Sleep may also allow for the reallocation of energy to other processes (Lesku & Schmidt, 2022; Preston et al., 2009; Schmidt, 2014). Arabian oryx (*Oryx leucoryx*) sleep during the night in winter, but switch to sleeping mid‐day in summer, perhaps to reduce the thermoregulatory costs of being active under the hot Saudi Arabian sun (Davimes et al., 2018).

**Figure 1:**
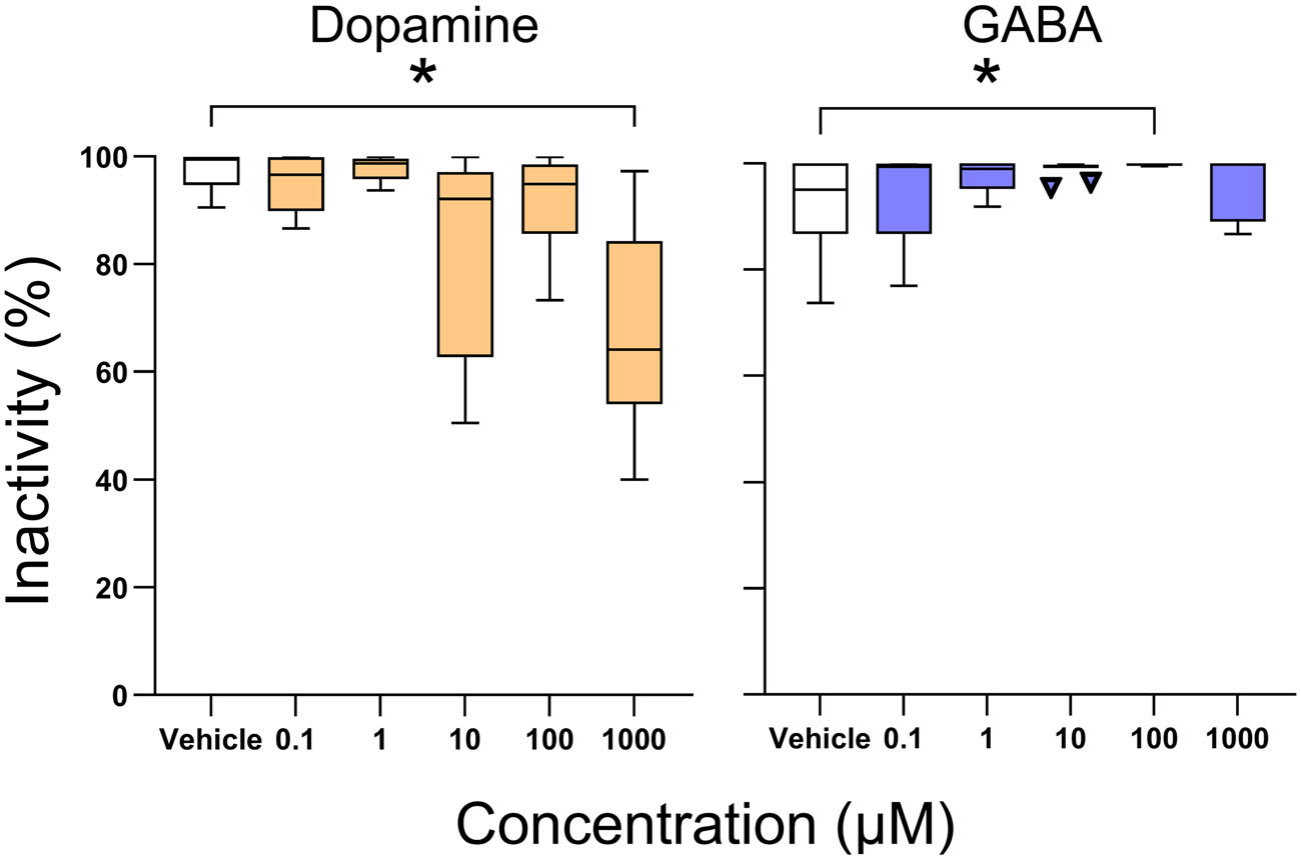
Effects of dopamine and GABA on the amount of restfulness in flatworms. Owing to the observation that dopamine reduced inactivity at 1000 µM and GABA increased inactivity at 100 µM, we chose these concentrations for the present study. The inverted U‐shaped dose–response curve for GABA is common in pharmacological experiments (Calabrese & Baldwin, 2003). Data modified from Omond et al. (2022). GABA, gamma‐aminobutyric acid.

Flatworms are among the simplest animals. Lacking a circulatory system, respiratory system, endocrine glands, a coelom, and anus, flatworms nonetheless possess a central and peripheral nervous system and various sensory systems (Ruppert et al., 2004). Importantly, restful flatworms have been shown to be asleep as evidenced by postural changes, reduced responsiveness, and rebound following sleep loss (Bounas et al., 2024; Omond et al., 2017). Furthermore, when given dopamine, a neurotransmitter that often regulates wakefulness (Joiner, 2016; Stenberg, 2007), flatworms move more and move farther (Figure 1) (Omond et al., 2022). Conversely, when given gamma‐aminobutyric acid (or GABA), an inhibitory neurotransmitter that puts vertebrates, flies, and even *Hydra* to sleep (Kanaya et al., 2020; Winsky‐Sommerer, 2009), flatworms are more restful suggesting that they, too, fall asleep from GABA (Omond et al., 2022).

Here, we investigated whether the effects of neurotransmitters on flatworm behavior reflect underlying changes to their physiological state. To do so, we administered dopamine or GABA to flatworms, in addition to having day‐time and night‐time neurotransmitter‐free controls. Under a strong hypothesis‐testing framework, we tested four predictions. Oxygen consumption rate (OCR) would be: (*1*) higher at night, when flatworms are most active, relative to the day‐time, (*2*) higher still with excitatory dopamine, and (*3*) lower with inhibitory GABA, relative to controls, and (*4*) lower with GABA compared to animals dosed with dopamine.

## 2 METHOD

Free‐living flatworms (*Girardia tigrina*, Girard, 1850) were wild‐caught by Southern Biological (Melbourne, Australia) and housed at La Trobe University. The flatworms were maintained on a 12:12 light:dark photoperiod (lights off at 2000 h), in a temperature‐controlled room at 19 ± 2°C. On arrival, the flatworms were separated into control and treatment groups, housed in ceramic bowls (15 cm diameter), and fed hard‐boiled egg yolk twice weekly. After 14‐days of habitation to these laboratory conditions, the animals were fed and then fasted 14 days before the experiment. This level of food restriction may seem severe, yet these simple ectothermic invertebrates handle this step without ill effect (Omond et al., 2022). Water was changed twice weekly. Data was collected between 0800 and 1200 h for the day‐time trials (control and dopamine), and 2000−0000 h for the night‐time trials (control and GABA). All animals were used once, and animals tested between 2000 and 0000 h were kept under dim red light postfeeding, a wavelength of light that flatworms cannot see (Paskin et al., 2014).

**Figure 2:**
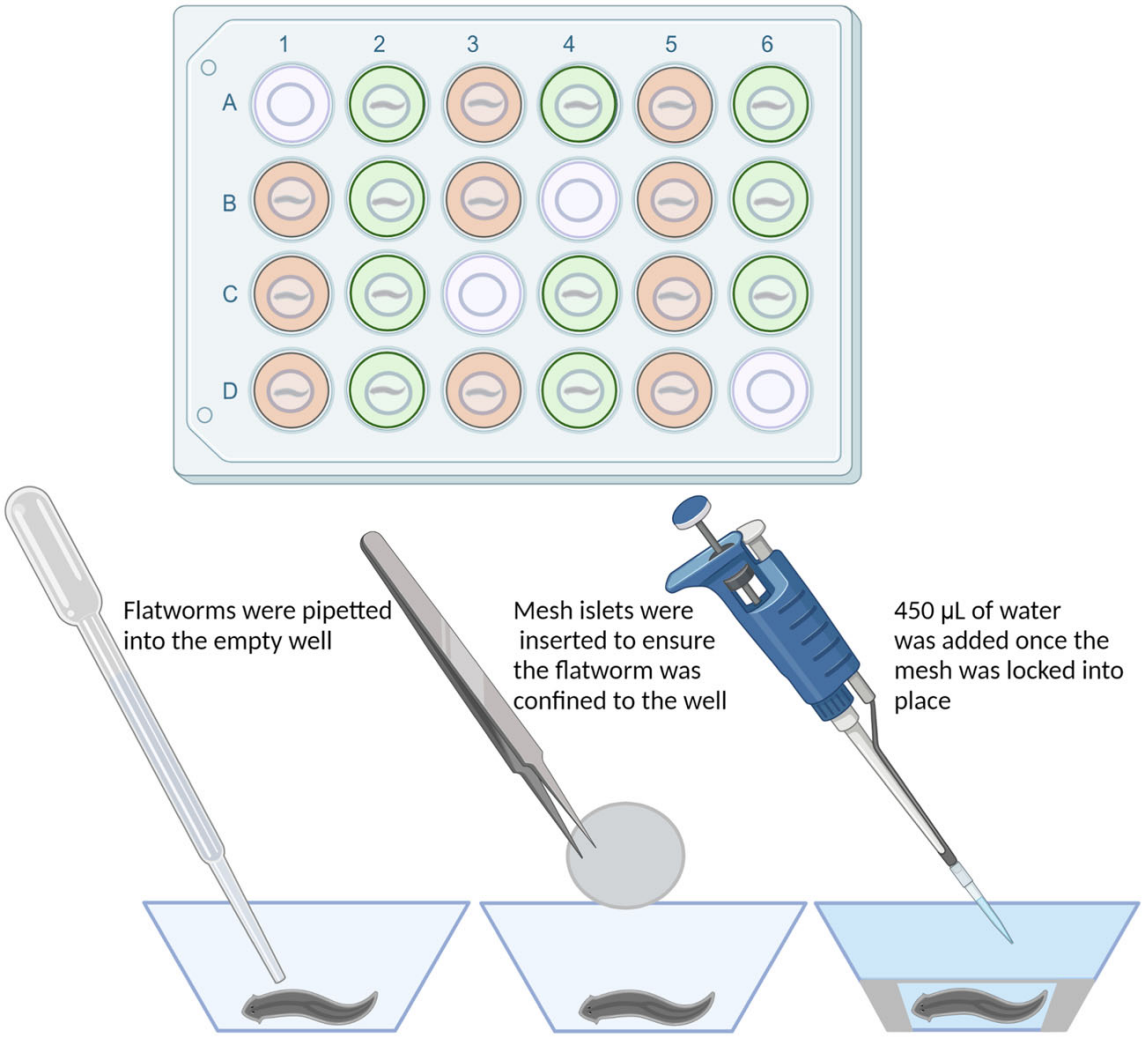
Overview of plate loading and organization of neurotransmitters (orange wells), neurotransmitter‐free controls (green), and water‐only blanks (blue) within the 24‐well Seahorse plate (top). The spatial organization of dopamine and GABA were alternated between trials. Below the plate is a step‐by‐step description of adding flatworms, and the islet mesh into each well. Image created using BioRender.com.

At the beginning of a trial, animals were divided into one of four treatments. Flatworms were offered a hard‐boiled egg yolk imbued with neurotransmitter, while themselves “bathing” in one of: 1000 µM dopamine (*n* = 20), 100 µM GABA (*n* = 29), or neurotransmitter‐free spring water control during the day‐time (*n* = 28) or night‐time (*n* = 27), following the methods established by Omond et al. (2022). Dopamine (Cat. H8502) and GABA (Cat. A2129) were purchased from Sigma‐Aldrich Pty Ltd and reconstituted from powder (see Omond et al., 2022). Feeding (and bathing) lasted 40 min, after which the animals were deposited, one per well (following Osuma et al., 2018), into a 24‐well Islet capture microplate along with 50 µL of their respective treatment solution; a Seahorse XFe24 mesh islet was placed into each well to prevent the flatworms climbing out (Figure 2). Once the islet was in place, an additional 450 µL of treatment‐specific solution was added. The flatworms could move very little in the well. Four wells remained empty, but for spring water, to serve as flatworm‐free controls. The 24‐well plate was then inserted into the Seahorse XFe24 (Agilent Technologies). The protocol for measurements of OCR was 15 rounds of: 60 s mix, 30 s wait, and 120 s measurement for a total experimental time of 52.5 min. Specifically, during the measurement phase, the probe assembly is lowered into the wells of the plate, where it seals each well to make a small, transient microchamber (approximately 7 µL). As the worm consumes oxygen, the levels drop. However, through mixing, the microchamber is reoxygenated, ensuring the samples do not become hypoxic. To mix, the probe assembly is repeatedly lowered into, and raised from, the plate/wells and that process allows oxygen to rediffuse into the chamber liquid. A period of wait time is incorporated to allow the samples to settle before the commencement of the next measurement cycle. The default settings of the respirometer are 180 s mix, 60 s wait, and 180 s measure. We shortened each of these times to minimize physiological stress. For instance, a longer mix time might stress or harm the flatworms by the descending probe; a longer measurement time might lead to hypoxia in the microchamber. The heater in the Seahorse unit was turned off, and yet over the course of the 52.5‐min trial, well temperatures increased modestly by 0.5–0.7°C, with starting temperatures ranging from 21.5°C to 23.2°C. Six plates were needed to generate a statistically‐meaningful sample size.

After the 52.5 min cycle measuring OCR, animals were photographed using an Olympus SZ61 microscope to ensure that they had eaten the egg yolk and were still alive. Animals that had not eaten, and deceased flatworms, were excluded from further analysis. Once the photos had been taken, flatworms were placed into 2 µL of Milli‐Q water and stored for later total protein quantification at −80°C. When measuring metabolic rate, it is important to statistically control for body mass. An attempt at weighing the flatworms proved too variable (data not shown), and so we opted to measure the amount of protein per flatworm as a proxy for body mass. Total protein analysis was conducted by adding 100 µL of lysis buffer (2% SDS, 50 mM NH_4_HCO_3_), and vortexing each flatworm. Now‐deceased animals rested in the buffer for 30 min before 400 µL of Milli‐Q water was added and the flatworms were vortexed again. Worms were centrifuged at 10.000*g* for 120 s. One µL of the flatworm lysate was aliquoted in a Qubit® (Invitrogen™, ThermoFisher), along with 198 µL of buffer and 1 µL of dye from the Qubit® Quant‐iT protein assay kit. The tube was then incubated for 15 min before being placed into the Qubit® fluorometer; total protein was analyzed as µg/mL.

Data was analyzed using analysis of covariance looking at the main effect of treatment, while holding constant the effects of body size (i.e., protein content) and temperature. Statistical analyses were performed in SYSTAT 13 (SPSS, Inc.).

## 3 RESULTS

We first predicted that nocturnal flatworms would have a higher OCR at night relative to the day‐time, while controlling for any effects of body size or temperature. Although flatworms with more protein did have a higher OCR, OCR did not differ by the time‐of‐day (Table 1). Next, we predicted that OCR would be higher for flatworms dosed with excitatory dopamine compared to the day or night controls. Neither of these predictions were strongly supported by the data; however, there was a trend for OCR to be higher in dopamine‐treated flatworms relative to the night‐time control (*p* = 0.057). Third, we predicted that OCR would be lower between animals given inhibitory GABA and the night and day controls. Here, we found mixed support. GABA did not lower OCR relative to the night‐time control, but did so compared to the day‐time vehicle. Lastly, we expected that OCR would be different between animals dosed with each of the neurotransmitters. Accordingly, OCR was lower with GABA relative to dopamine.

**Table 1:** Results of six analyses of covariance (ANCOVA) to test the predictions set out in the Introduction.

Comparison	Factor	SS	*df*	MS	*F* ratio	*p* Value
Day versus night	Treatment	43,282	1	43,282	2.026	0.161
	Protein	319,569	1	319,569	14.957	<0.001
	Temperature	542	1	542	0.025	0.874
	Error	1,089,675	51	21,366		
Day versus dopamine	Treatment	19,784	1	19,784	1.125	0.295
	Protein	567,763	1	567,763	32.285	<0.001
	Temperature	9883	1	9883	0.562	0.457
	Error	773,777	44	17,586		
Night versus dopamine	Treatment	82,514	1	82,514	3.813	0.057
	Protein	343,034	1	343,034	15.853	<0.001
	Temperature	186,357	1	186,357	8.613	0.005
	Error	930,430	43	21,638		
Night versus GABA	Treatment	14,300	1	14,300	0.671	0.416
	Protein	293,665	1	293,665	13.777	0.001
	Temperature	72,448	1	72,448	3.399	0.071
	Error	1,108,438	52	21,316		
Day versus GABA	Treatment	100,064	1	100,064	5.574	0.022
	Protein	465,516	1	465,516	25.932	<0.001
	Temperature	293	1	293	0.016	0.899
	Error	951,418	53	17,951		
Dopamine versus GABA	Treatment	160,776	1	160,776	10.688	0.002
	Protein	377,611	1	377,611	25.103	<0.001
	Temperature	193,628	1	193,628	12.872	0.001
	Error	676,915	45	15,043		

*Note*: The factor *treatment* refers to the two variables being compared as specified in the first column; protein is the amount of protein per flatworm (a proxy for the size of each animal); *temperature* is the temperature inside the Seahorse respirometer unit. In each ANCOVA, flatworms with more protein had a high oxygen consumption rate (*p* ≤ 0.001); the effect of temperature was nonsignificant in four of six ANCOVAs. Importantly, flatworms given GABA had a significantly lower metabolic rate compared to the day‐time control and those given dopamine (shaded rows); there was a trend for dopamine to increase oxygen consumption relative to the night‐time control.

Abbreviations: ANCOVA, analysis of covariance; GABA, gamma‐aminobutyric acid.

**Figure 3:**
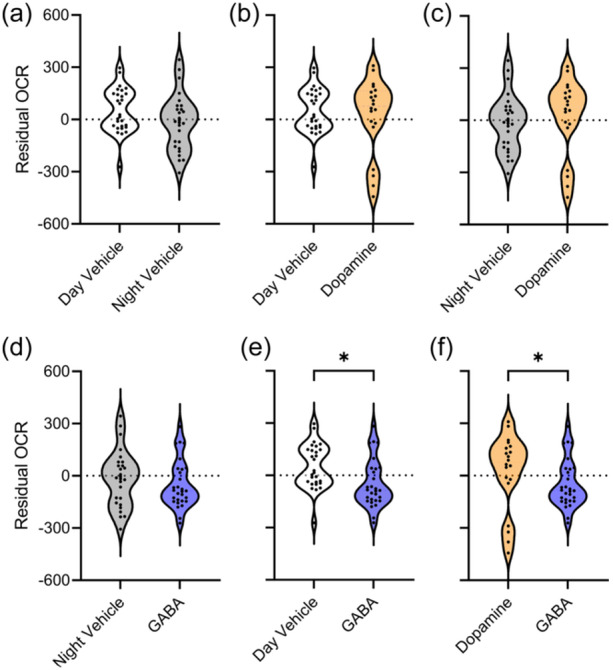
Violin plots of residual oxygen consumption rate (OCR), that controls for the size of each flatworm, during (a) day‐ and night‐time neurotransmitter‐free controls, (b) day‐time control and dopamine, (c) night‐time control and dopamine, (d) night‐time control and GABA, (e) day‐time control and GABA, and (f) dopamine and GABA. The width of the shaded area in each panel reflects the proportion of data located there. Statistical significance (*) is taken from Table 1 (*p* < 0.050). GABA, gamma‐aminobutyric acid.

Another way to view the data, other than a statistical table, is a bivariate plot between metabolic rate and the two groups being considered. But, plotting multivariate data on two axes poses an obvious problem. To overcome this, we needed a single metabolic rate value that also controlled for body size. And so, we extracted the residuals from a linear regression of total protein (µg/mL) against the OCR (pmol/min). The resulting “residual OCR” variable controlled for variation in body size (as per Kelly et al., 2022; Lesku et al., 2009) and could be used as a visual approximation of the results in Table 1 (Figure 3).

## 4 DISCUSSION

The neurotransmitters altered oxygen consumption in flatworms. Notably, GABA, which causes flatworms to become behaviorally restful (Omond et al., 2022), decreased oxygen consumption relative to both the day‐time control and to animals dosed with dopamine. These reduced metabolic demands agree with Keenan et al. (1979) who demonstrated that GABA suppressed neuronal activity in the nerve cords of another flatworm (*Notoplana acticola*). Furthermore, GABA promotes sleep across distantly related animals, from vertebrates to the cnidarian *Hydra* (Kanaya et al., 2020; Stenberg, 2007; Winsky‐Sommerer, 2009). Taken together, these results suggest that GABA induces sleep in flatworms. That said, it is important to evaluate this interpretation using other measures of sleep, such as arousal thresholds (Omond et al., 2017) and electrophysiology (Freiberg et al., 2023).

Studies of metabolism in fruit flies found that metabolic rate decreased at sleep onset and more so as the sleep bout progressed (Stahl et al., 2017). Stahl et al. (2017) also showed an increase in metabolic rate in animals during the day‐time; however, those that had been dosed with the GABA agonist gaboxadol had lower metabolic rates than control animals, in accordance with GABA reducing metabolism in flatworms. Another study looked at dopamine transport (DAT)‐defective fruit fly mutants, *fumin* (*fmn*), which are short sleepers (Ueno et al., 2012). These animals have a normal circadian activity rhythm, but exhibit a different profile than wild‐type flies. Ueno et al. (2012) showed that fruit flies that had higher circulating levels of dopamine also had higher metabolic rates compared to wild‐type flies, a pattern that persisted in *fmn* flies that did not have increased locomotor activity. Taken together, the findings of Stahl et al. (2017) and Ueno et al. (2012) are consistent with our result that flatworms dosed with GABA have a lower metabolic rate than those dosed with dopamine, and justify also the trend for dopamine‐treated flatworms to have a higher OCR than the night‐time controls.

Surprisingly, our other predictions were not supported by the data. Dopamine did not significantly augment OCR relative to the controls. Perhaps this is simply because of the lower sample size for dopamine relative to the other treatments. It could also arise from the environment within the Seahorse respirometer, or our feeding protocol. First, while undisturbed flatworms sleep during the day‐time, it seems likely they were not sleeping in the respirometer. During the recordings, the Seahorse unit has mixing and waiting phases, along with a (near) absence of light. During the measurement phase, the Seahorse uses its red (650 nm), green (530 nm), and blue (470 nm) light emitting diodes to obtain the oxygen reading (Agilent, 2018). When exposed to shorter wavelengths of light, planaria avoid lit areas (Paskin et al., 2014); it is unknown whether the low intensity of light within the Seahorse is visible to flatworms. Furthermore, electrical activity in flatworms appears to increase under bright light (Freiberg et al., 2023). The lights of the Seahorse unit may have stimulated the negatively‐phototactic flatworms, and increased oxygen consumption in the controls, masking any effect of dopamine. It is also possible that feeding flatworms before measurements increased metabolic rate owing to specific dynamic action, that might have masked an effect of dopamine increasing OCR. These explanations might also explain the similarity in oxygen consumption between day‐time and night‐time controls; for instance, if the respirometer environment, or pre‐measurement feeding, caused metabolic rates to be heightened.

Metabolic rate is often temperature‐dependent, particularly in ectotherms. In *Cassiopea* jellyfish, metabolic rate is higher during the day and increases with temperature (Aljbour et al., 2017; Welsh et al., 2009). Similarly, sea sponges (*Aplysina aerophoba*, *Grantia* spp., *Geodia barretti*, *Negombata magnifica*) have elevated metabolic rates with warmer temperatures (Hadas et al., 2008; Hoffmann et al., 2008; Hyman, 1925; Strand et al., 2017). We did not find the metabolic rate of flatworms to be consistently dependent on ambient temperature; however, it is important to remember that our temperature variability was less than 1°.

One avenue for future research is the use of electrophysiology to probe the physiology of the flatworm brain. The sleeping brain of invertebrates appears to often have lower activity relative to one awake (Bushey et al., 2015; Kaiser & Steiner‐Kaiser, 1983; Nitz et al., 2002). And so, we expect that sleeping flatworms, and flatworms exposed to GABA, would have less brain activity (or lower spectral power density) than awake flatworms and those exposed to dopamine. With the innovation of nonlethal electrophysiological recording techniques (Freiberg et al., 2023), such studies are now possible. Overall, this is the first study to look at how time‐of‐day and neurotransmitters impact the OCR of flatworms. While flatworms are known to sleep, it had been unclear whether restful flatworms reap the metabolic benefits of this quiescent state. Using neurotransmitters that regulate sleep and wakefulness, we show that sleep‐promoting GABA reduces oxygen consumption in flatworms.

## CONFLICT OF INTEREST STATEMENT

The authors declare no conflict of interest.

## Data Availability

The data that support the findings of this study are available from the corresponding author upon reasonable request.

## References

[jez2828-bib-0001] Agilent . (2018). Seahorse XFe Analyzer: Operating Manual. Agilent Technologies.

[jez2828-bib-0002] Aljbour, S. M. , Zimmer, M. , & Kunzmann, A. (2017). Cellular respiration, oxygen consumption, and trade‐offs of the jellyfish *Cassiopea* sp. in response to temperature change. Journal of Sea Research, 128, 92–97.

[jez2828-bib-0003] Berger, R. J. , & Phillips, N. H. (1995). Energy conservation and sleep. Behavioural Brain Research, 69, 65–73. 7546319 10.1016/0166-4328(95)00002-b

[jez2828-bib-0004] Bounas, A. , Komini, C. , Toli, E. A. , Talioura, A. , Sotiropoulos, K. , & Barboutis, C. (2024). Expression patterns of heat‐shock genes during stopover and the trade‐off between refueling and stress response in a passerine migrant. Journal of Comparative Physiology B, 194, 1–6. 10.1007/s00360-023-01529-x PMC10940366 38296861

[jez2828-bib-0005] Brown, E. B. , Klok, J. , & Keene, A. C. (2022). Measuring metabolic rate in single flies during sleep and waking states via indirect calorimetry. Journal of Neuroscience Methods, 376, 109606. 35483506 10.1016/j.jneumeth.2022.109606 PMC9310448

[jez2828-bib-0006] Bushey, D. , Tononi, G. , & Cirelli, C. (2015). Sleep‐ and wake‐dependent changes in neuronal activity and reactivity demonstrated in fly neurons using in vivo calcium imaging. Proceedings of the National Academy of Sciences, 112, 4785–4790. 10.1073/pnas.1419603112 PMC4403205 25825756

[jez2828-bib-0007] Calabrese, E. J. , & Baldwin, L. A. (2003). Hormesis: The dose‐response revolution. Annual Review of Pharmacology and Toxicology, 43, 175–197. 10.1146/annurev.pharmtox.43.100901.140223 12195028

[jez2828-bib-0008] Davimes, J. G. , Alagaili, A. N. , Bhagwandin, A. , Bertelsen, M. F. , Mohammed, O. B. , Bennett, N. C. , Manger, P. R. , & Gravett, N. (2018). Seasonal variations in sleep of free‐ranging Arabian oryx (*Oryx leucoryx*) under natural hyperarid conditions. Sleep, 41, zsy038. 10.1093/sleep/zsy038 29474674

[jez2828-bib-0009] Ferretti, A. , Rattenborg, N. C. , Ruf, T. , McWilliams, S. R. , Cardinale, M. , & Fusani, L. (2019). Sleeping unsafely tucked in to conserve energy in a nocturnal migratory songbird. Current Biology, 29, 2766–2772.e4. 31430467 10.1016/j.cub.2019.07.028

[jez2828-bib-0010] Freiberg, J. , Lang, L. , Kaernbach, C. , & Keil, J. (2023). Characterization of the planarian surface electroencephalogram. BMC Neuroscience, 24, 29. 37138236 10.1186/s12868-023-00799-z PMC10157967

[jez2828-bib-0011] Hadas, E. , Ilan, M. , & Shpigel, M. (2008). Oxygen consumption by a coral reef sponge. Journal of Experimental Biology, 211, 2185–2190. 18552308 10.1242/jeb.015420

[jez2828-bib-0012] Hoffmann, F. , Røy, H. , Bayer, K. , Hentschel, U. , Pfannkuchen, M. , Brümmer, F. , & de Beer, D. (2008). Oxygen dynamics and transport in the Mediterranean sponge *Aplysina aerophoba* . Marine Biology, 153, 1257–1264. 24391232 10.1007/s00227-008-0905-3 PMC3873076

[jez2828-bib-0013] Hyman, L. H. (1925). Respiratory differences along the axis of the sponge *Grantia* . The Biological Bulletin, 48, 379–389.

[jez2828-bib-0014] Joiner, W. J. (2016). Unraveling the evolutionary determinants of sleep. Current Biology, 26, R1073–R1087. 27780049 10.1016/j.cub.2016.08.068 PMC5120870

[jez2828-bib-0015] Kaiser, W. , & Steiner‐Kaiser, J. (1983). Neuronal correlates of sleep, wakefulness and arousal in a diurnal insect. Nature, 301, 707–709. 6828153 10.1038/301707a0

[jez2828-bib-0016] Kanaya, H. J. , Park, S. , Kim, J. , Kusumi, J. , Krenenou, S. , Sawatari, E. , Sato, A. , Lee, J. , Bang, H. , Kobayakawa, Y. , Lim, C. , & Itoh, T. Q. (2020). A sleep‐like state in *Hydra* unravels conserved sleep mechanisms during the evolutionary development of the central nervous system. Science Advances, 6, eabb9415. 33028524 10.1126/sciadv.abb9415 PMC7541080

[jez2828-bib-0017] Keenan, L. , Koopowitz, H. , & Bernardo, K. (1979). Primitive nervous systems: action of aminergic drugs and blocking agents on activity in the ventral nerve cord of the flatworm *Notoplana acticola* . Journal of Neurobiology, 10, 397–407. 38300 10.1002/neu.480100406

[jez2828-bib-0018] Kelly, M. L. , Collins, S. P. , Lesku, J. A. , Hemmi, J. M. , Collin, S. P. , & Radford, C. A. (2022). Energy conservation characterizes sleep in sharks. Biology Letters, 18, 20210259. 35259943 10.1098/rsbl.2021.0259 PMC8915397

[jez2828-bib-0019] Lesku, J. A. , Roth II, T. C. , Rattenborg, N. C. , Amlaner, C. J. , & Lima, S. L. (2009). History and future of comparative analyses in sleep research. Neuroscience & Biobehavioral Reviews, 33, 1024–1036. 19443034 10.1016/j.neubiorev.2009.04.002

[jez2828-bib-0020] Lesku, J. A. , & Schmidt, M. H. (2022). Energetic costs and benefits of sleep. Current Biology, 32, R656–R661. 35728548 10.1016/j.cub.2022.04.004

[jez2828-bib-0021] Lima, S. L. , Rattenborg, N. C. , Lesku, J. A. , & Amlaner, C. J. (2005). Sleeping under the risk of predation. Animal Behaviour, 70, 723–736.

[jez2828-bib-0022] Nitz, D. A. , van Swinderen, B. , Tononi, G. , & Greenspan, R. J. (2002). Electrophysiological correlates of rest and activity in *Drosophila melanogaster* . Current Biology, 12, 1934–1940. 12445387 10.1016/s0960-9822(02)01300-3

[jez2828-bib-0023] Omond, S. , Ly, L. M. T. , Beaton, R. , Storm, J. J. , Hale, M. W. , & Lesku, J. A. (2017). Inactivity is nycthemeral, endogenously generated, homeostatically regulated, and melatonin modulated in a free‐living platyhelminth flatworm. Sleep, 40, zsx124. 10.1093/sleep/zsx124 28958003

[jez2828-bib-0024] Omond, S. E. T. , Hale, M. W. , & Lesku, J. A. (2022). Neurotransmitters of sleep and wakefulness in flatworms. Sleep, 45, zsac053. 35554581 10.1093/sleep/zsac053 PMC9216492

[jez2828-bib-0025] Osuma, E. A. , Riggs, D. W. , Gibb, A. A. , & Hill, B. G. (2018). High throughput measurement of metabolism in planarians reveals activation of glycolysis during regeneration. Regeneration, 5, 78–86. 29721328 10.1002/reg2.95 PMC5911454

[jez2828-bib-0026] Paskin, T. R. , Jellies, J. , Bacher, J. , & Beane, W. S. (2014). Planarian phototactic assay reveals differential behavioral responses based on wavelength. PLoS One, 9, e114708. 25493551 10.1371/journal.pone.0114708 PMC4262426

[jez2828-bib-0027] Preston, B. T. , Capellini, I. , McNamara, P. , Barton, R. A. , & Nunn, C. L. (2009). Parasite resistance and the adaptive significance of sleep. BMC Evolutionary Biology, 9, 7. 19134175 10.1186/1471-2148-9-7 PMC2631508

[jez2828-bib-0028] Rattenborg, N. C. , & Ungurean, G. (2023). The evolution and diversification of sleep. Trends in Ecology & Evolution, 38, 156–170. 36411158 10.1016/j.tree.2022.10.004

[jez2828-bib-0029] Ruppert, E. E. , Fox, R. S. , & Barnes, R. D. (2004). Invertebrate Zoology: A Functional Evolutionary Approach (7th ed.). Thomson, Brooks/Cole.

[jez2828-bib-0030] Schmidt, M. H. (2014). The energy allocation function of sleep: A unifying theory of sleep, torpor, and continuous wakefulness. Neuroscience & Biobehavioral Reviews, 47, 122–153. 25117535 10.1016/j.neubiorev.2014.08.001

[jez2828-bib-0031] Sharma, S. , & Kavuru, M. (2010). Sleep and metabolism: An overview. International Journal of Endocrinology, 2010, 1–12. 10.1155/2010/270832 PMC2929498 20811596

[jez2828-bib-0032] Siegel, J. M. (2005). Clues to the functions of mammalian sleep. Nature, 437, 1264–1271. 16251951 10.1038/nature04285 PMC8760626

[jez2828-bib-0033] Siegel, J. M. (2009). Sleep viewed as a state of adaptive inactivity. Nature Reviews Neuroscience, 10, 747–753. 19654581 10.1038/nrn2697 PMC8740608

[jez2828-bib-0034] Stahl, B. A. , Slocumb, M. E. , Chaitin, H. , DiAngelo, J. R. , & Keene, A. C. (2017). Sleep‐dependent modulation of metabolic rate in *Drosophila* . Sleep, 40, zsx084. 28541527 10.1093/sleep/zsx084 PMC6074949

[jez2828-bib-0035] Stenberg, D. (2007). Neuroanatomy and neurochemistry of sleep. Cellular and Molecular Life Sciences, 64, 1187–1204. 17364141 10.1007/s00018-007-6530-3 PMC11136155

[jez2828-bib-0036] Strand, R. , Whalan, S. , Webster, N. S. , Kutti, T. , Fang, J. K. H. , Luter, H. M. , & Bannister, R. J. (2017). The response of a boreal deep‐sea sponge holobiont to acute thermal stress. Scientific Reports, 7, 1660. 28533520 10.1038/s41598-017-01091-x PMC5440399

[jez2828-bib-0037] Ueno, T. , Tomita, J. , Kume, S. , & Kume, K. (2012). Dopamine modulates metabolic rate and temperature sensitivity in *Drosophila melanogaster* . PLoS One, 7, e31513. 22347491 10.1371/journal.pone.0031513 PMC3274542

[jez2828-bib-0038] Welsh, D. T. , Dunn, R. J. K. , & Meziane, T. (2009). Oxygen and nutrient dynamics of the upside down jellyfish (*Cassiopea* sp.) and its influence on benthic nutrient exchanges and primary production. Hydrobiologia, 635, 351–362.

[jez2828-bib-0039] Winsky‐Sommerer, R. (2009). Role of GABA_A_ receptors in the physiology and pharmacology of sleep. European Journal of Neuroscience, 29, 1779–1794. 19473233 10.1111/j.1460-9568.2009.06716.x

[jez2828-bib-0040] Zaid, E. , Rainsford, F. W. , Johnsson, R. D. , Valcu, M. , Vyssotski, A. L. , Meerlo, P. , & Lesku, J. A. (2024). Semelparous marsupials reduce sleep for sex. Current Biology, 34, 606–614.e3. 38278151 10.1016/j.cub.2023.12.064

[jez2828-bib-0041] Zepelin, H. , & Rechtschaffen, A. (1974). Mammalian sleep, longevity, and energy metabolism. Brain, Behavior and Evolution, 10, 425–446. 4464027 10.1159/000124330

